# A Nightmare’s Lullaby: Exploring the concept and relevance of oneiroid cyclic psychosis through a clinical case and review

**DOI:** 10.1192/j.eurpsy.2022.1827

**Published:** 2022-09-01

**Authors:** A. Compaired Sánchez

**Affiliations:** Hospital Universitario Ramón y Cajal, Psychiatry, Madrid, Spain

**Keywords:** oneiroid syndrome, Mood disorders, biological-rhythm dysregulation, cyclic psychosis

## Abstract

**Introduction:**

Acute psychotic states characterized by clinical lability and dream-like qualities are a staple of classic psychopatology. An excessive focus on diagnostic criteria for bipolar or schizophrenia-spectrum disorders risks missing this particular set of patients; defined through their dynamic presentation as much as by any cluster of symptoms or types of course.

**Objectives:**

To explore the concept and relevance of oneroid-like cyclic psychosis through a clinical case and review.

**Methods:**

We report the case of a 37 year old woman with bipolar disorder (three previous instances of manic episodes with psychotic symptoms) and various gynecological issues that required hormone therapy. After a couple of days having difficulty sleeping, the patient developed a clinical picture consisting of wide and sudden oscillations between hyperactive and inhibited psychomotor activity, moods of dread and ecstasy, and states of disorganized thought and childlike activities with perplexity and mutism. Frequent behaviors as if experiencing visual alucinations and repeated allusions to feeling as if in a dream. These symptoms lasted for 2-3 weeks, after treatment with risperidone and lithium. A narrative review concerning the case was also performed.

**Results:**

Kleist’s ‘innate instability’ permeates much of the previous literature. Similar entities highlight different issues closely related to various biological rhythms: atypical psychosis and epilepsy, puerperal psychosis and estrogen dysregulation, cyclic psychosis and sleep disorders, delirious mania and effectiveness of electro-convulsive-therapy, etc.

**Conclusions:**

Our findings point to the clinical relevance of oneiroid cyclic psychosis as innate instability. Further studies on the role of biological rhythms and its repercussions on daily practice are required.

**Disclosure:**

No significant relationships.

